# Experimental and numerical investigation of polymer pore-clogging in micromodels

**DOI:** 10.1038/s41598-023-34952-9

**Published:** 2023-05-22

**Authors:** Antonia Sugar, Maged Serag, Ulrich Buttner, Marwan Fahs, Satoshi Habuchi, Hussein Hoteit

**Affiliations:** 1 Physical Science and Engineering Division, King Abdullah University of Science and Technology (KAUST), Thuwal, Saudi Arabia; 2 Biological and Environmental Science and Engineering Division, King Abdullah University of Science and Technology, Thuwal, Saudi Arabia; 3 Nanofabrication Core Lab, King Abdullah University of Science and Technology, Thuwal, Saudi Arabia; 4 Institut Terre et Environnement de Strasbourg, Université de Strasbourg, CNRS, ENGEES, Strasbourg, France

**Keywords:** Hydrology, Geodynamics, Hydrogeology

## Abstract

Polymers have been used effectively in the Oil & Gas Industry for a variety of field applications, such as enhanced oil recovery (EOR), well conformance, mobility control, and others. Polymer intermolecular interactions with the porous rock, in particular, formation clogging and the associated alterations to permeability, is a common problem in the industry. In this work, fluorescent polymers and single-molecule imaging are presented for the first time to assess the dynamic interaction and transport behavior of polymer molecules utilizing a microfluidic device. Pore-scale simulations are performed to replicate the experimental observations. The microfluidic chip, also known as a "Reservoir-on-a-Chip" functions as a 2D surrogate to evaluate the flow processes that take place at the pore-scale. The pore-throat sizes of an oil-bearing reservoir rock, which range from 2 to 10 nm, are taken into consideration while designing the microfluidic chip. Using soft lithography, we created the micromodel from polydimethylsiloxane (PDMS). The conventional use of tracers to monitor polymers has a restriction due to the tendency of polymer and tracer molecules to segregate. For the first time, we develop a novel microscopy method to observe the dynamic behavior of polymer pore-clogging and unclogging processes. We provide direct dynamic observations of polymer molecules during their transport within the aqueous phase and their clustering and accumulations. Pore-scale simulations were carried out to simulate the phenomena using a finite-element simulation tool. The simulations revealed a decline in flow conductivity over time within the flow channels that experienced polymer accumulation and retention, which is consistent with the experimental observation of polymer retention. The performed single-phase flow simulations allowed us to assess the flow behavior of the tagged polymer molecules within the aqueous phase. Additionally, both experimental observation and numerical simulations are used to evaluate the retention mechanisms that emerge during flow and how they affect apparent permeability. This work provides new insights to assessing the mechanisms of polymer retention in porous media.

## Introduction

Polymer flooding is a promising technique to chemically enhance oil recovery from conventional reservoirs^1,2^. The method implies the addition of water-soluble polymer molecules to the injection water to increase its phase viscosity. The viscosity enhancement leads to more favorable mobility ratios and water–oil fractional flow characteristics, which improves the sweep efficiency of the displacement process^3–5^. Even though polymer flooding is considered a mature EOR method, there are limited full-field implementations of polymer flooding^6^. This could be due to challenges of a successful implementation of the process at the field scale, from which polymer induced-formation damage is a major contributor^7–11^. Polymer-induced formation damage may impact the recovery performance by reducing the permeability of the formation and manifests as a continuous deterioration of well injectivity^12–15^. Polymer injectivity has been tied to water quality, incompatibility between injection and formation water, fine migration, among other factors^13,16^. The polymer-induced formation damage related to pore-throat clogging, or simply polymer clogging, is generated by the polymer entrapped in the pore space^17–21^.

Significant efforts have been devoted to study polymer transport behavior through porous media and polymer retention in reservoir rocks for decades^22–26^. Three main mechanisms are known to contribute to the overall retention of polymer during flow into porous rock, namely polymer adsorption, hydrodynamic retention, and mechanical entrapment^27,28^. But despite all the efforts, the mechanisms causing polymer entrapment are not fully understood. One of the reasons is that retention mechanisms are typically indirectly inferred from differential pressure and effluent concentration profiles during core-flooding experiments. Therefore, they fail to differentiate between different mechanisms and capture their individual effect on rocks^29–33^.

Therefore, a mechanistic understanding of polymer/pore-network interactions calls for a direct method to be studied. For this purpose, microfluidics represents a convenient platform, as it overcomes the constraints linked to the opacity of rocks^34,35^. Microfluidics hold promises to improve the understanding of fluid flow in porous formations by providing direct observation of the phenomena^36–39^. In recent years, microfluidics experiments have been increasingly used to address single-phase and multi-phase flow of polymer solutions through porous media. Some experimental studies have looked at the rheological characteristics of polymer solutions, flow instabilities, and flow divergence. Liu et al.^40^ conducted experiments on micromodels to study particle migration and clogging in porous media. Others focused on recovery factor, and sweep efficiency, among other applications^41–48^. Engl et al.^49^ used capillary tubes at microscale to study the transport of confined droplets at channel junctions.

Computational-fluid-dynamics (CFD) simulations are powerful tools to accurately simulate complex physical models and interactions. In the area where microfluidics emerged, in bioscience, numerical modeling of biological-related phenomena sees great advancements. Numerical studies incorporating multiple coupled particle-solid and particle–particle interactions are used to analyze experimental results on air-borne nanoparticle transport in lungs or blockage in arteries, or DNA clog and release^50–52^. However, in other research areas, simulations of fluid flow using microfluidics did not experience the same popularity as experimental studies of flow in micromodels. In the frame of EOR, the majority of these numerical studies target foam and microbial techniques, from which a significant proportion focus on the latter^53^. Jafari et al.^54^ performed CFD simulation of biosurfactant flooding into micromodels to evaluate the potential increase in oil production. In addition, oil displacement enhancement by bacteria suspension injection in micromodels has been studied both experimental and numerical^55^. Other studies investigated the influence of pore-scale hydrodynamics on the spatial evolution of bacterial biofilms^56^.

In the frame of polymer solution as a chemical method for EOR, limited numerical studies grounded on experiments are available in the literature. A study by^57^ investigated the viscous fingering reduction during polymer flooding compared to waterflooding and performed two-phase CFD simulations based on SEM images to replicate the experimental results. On the other hand, Afsharpoor et al. (2014) focused on the viscoelastic properties of polymer solution to assess the hypothesis of pulling-effects by an experimental investigation, complemented by CFD modeling to assess the flow characteristics of viscoelastic polymers in dead-end pores.

In this work, our objective is to further understand polymer transport and clogging mechanisms at the pore-scale by developing a numerical analysis approach to mimic the experimental observations^59^. We present a numerical study of polymer solution flow through a micromodel, performed using COMSOL Multiphysics®. A combined Lagrangian–Eulerian approach is used to model the flow and transport of polymer solution and agglomerates. A numerical model is proposed to qualitatively describe the clogging of polymer agglomerates and the resulting effect on flow divergence.

This paper is organized as follows: we first present the experimental data with the microfluidic device design and flow experiment results. Then, we introduce the equations that govern fluid and particle motion and briefly describe the simulation model. The results are then discussed, in which we demonstrate that the model replicates the flow behavior of polymer solution flow through the microdevice. The last part of the paper focuses on simulating polymer entrapment and the corresponding flow characteristics changes. We conclude the paper by summarizing the key contributions of the work.

## Experimental method and data

### Fabrication method

We use microfluidics technology coupled with a microscope setup to allow direct observations at pore- and molecule-scale under dynamic conditions. To enable visualization of the flow phenomena, the micromodels should be fully or partially optically transparent^60^. Microdevices materials include glass, silicon, polymeric materials, such as polydimethyl siloxane (PDMS) and polymethyl methacrylate (PMMA), geomaterials, and others^61^. Silicon is the primary material of microfluidic devices, which is a well-established fabrication method. The advantages of silicon-based microdevices include withstanding high pressure and temperature conditions, and compatible with many solvents^62,63^. The drawbacks of being opaque and high cost motivated the development of other alternative materials, such as glass and polymeric materials^64,65^. The most used elastomer for microdevice fabrication is polydimethylsiloxane (PDMS) (S. H. NK Karadimitriou 2012). The advantages of the PDMS-based microdevices are their optical transparency, flexibility, low price, and biocompatibility^60^.

**Figure 1:**
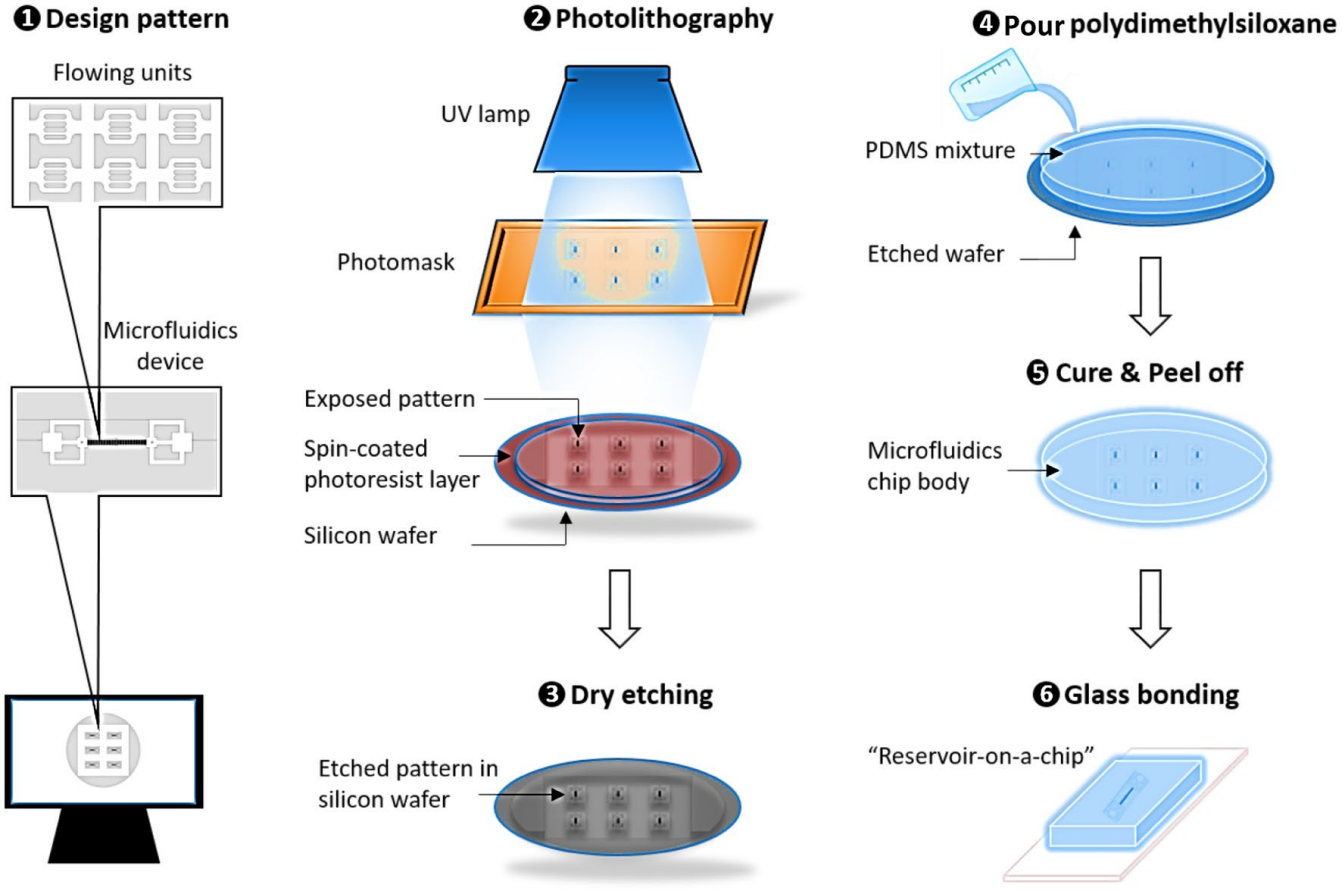
Workflow detailing the process of micromodel fabrication, starting with the (1) model design, followed by the (2) photolithography using a chrome mask, then (3) dry etching to engrave the pattern onto the silicon wafer, followed by (4) pouring the polydimethylsiloxane on the silicon wafer, then (5) curing and peel off, and finally (6) bonding the glass on top of the PDMS model.

In this work, we used the photolithography technique for fabricating silicon-based microdevices, with the actual pattern being etched into the silicon substrate and then bonded to the glass. Soft lithography is the main technique used for PDMS-based microdevices. With this technique, the polymer mixture is poured into a mold, holding the negative pattern, and then cured at an elevated temperature. The master mold used was silicon-based to generate increased accuracy on the small-sized features. We used a six-step fabrication workflow, as described in Fig. 1.

### Experimental data

**Figure 2:**
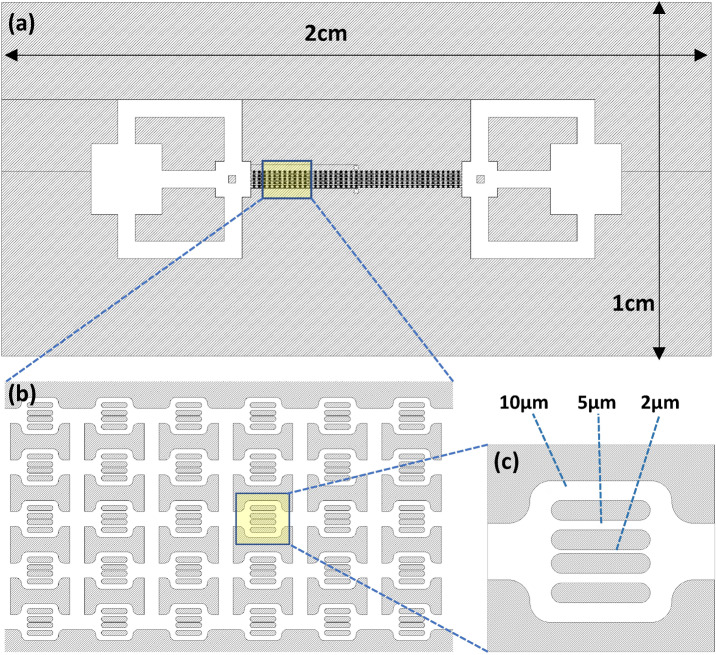
Top view of the entire microfluidic device of size 2 cm x1cm (**a**), including the configuration of the 40 × 5 flow units (**b**), and enlargement of the channels within each flow unit with dimensions 10, 5, and 2 μm (**c**).

The 2D micromodel design mimics key features related to the heterogeneous nature of rock pore throats while avoiding complexities related to the 3D nature of pore networks. Figure 2 shows a detailed schematic of the microfluidic device design. The structure consists of multiple flow units, which ensures repeatability and eliminates the end effects and the possibility of complete plugging of the chip. A flow unit is symmetric towards the horizontal axis and includes 2, 5, and 10 μm wide channels.

To minimize 3D effects, you used a microfluidic device with a height of 7 μm, a channel length (flow-unit) of almost 100 μm (see Fig. 2), and an overall model size of about 1 cm. This aspect ratio indicates that the height of the device is much smaller than its width and length, which would result in the flow being essentially 2D and not 3D. Moreover, the fluid flow in the microfluidic device is dominated by the frictional forces that occur at the channel wall, resulting in flow that is largely constrained to the plane of the channels, and any variations in the flow in the vertical direction are expected to be very small. Visual inspection in the experiment did not reveal any obvious 3D effects. This is likely because the 2D flow assumption was valid for the conditions of our experiment, and any small vertical variations in the flow did not have a significant effect on the overall flow behavior (see Video S1 in the supplementary material showing the dynamics of polymer transport and clogging in one flow unit). Nevertheless, it is important to note that in some other cases, the 3D effects may become important in microfluidic systems with larger aspect ratios or more complex geometries. Therefore, it is always important to carefully consider the appropriate assumptions and limitations of any model or experiment.

We fabricated the microdevice out of polydimethylsiloxane (PDMS) through soft-lithography techniques. We used an etched silicon wafer as a mold for the PDMS to increase the fabrication precision of the small features.

**Figure 3:**
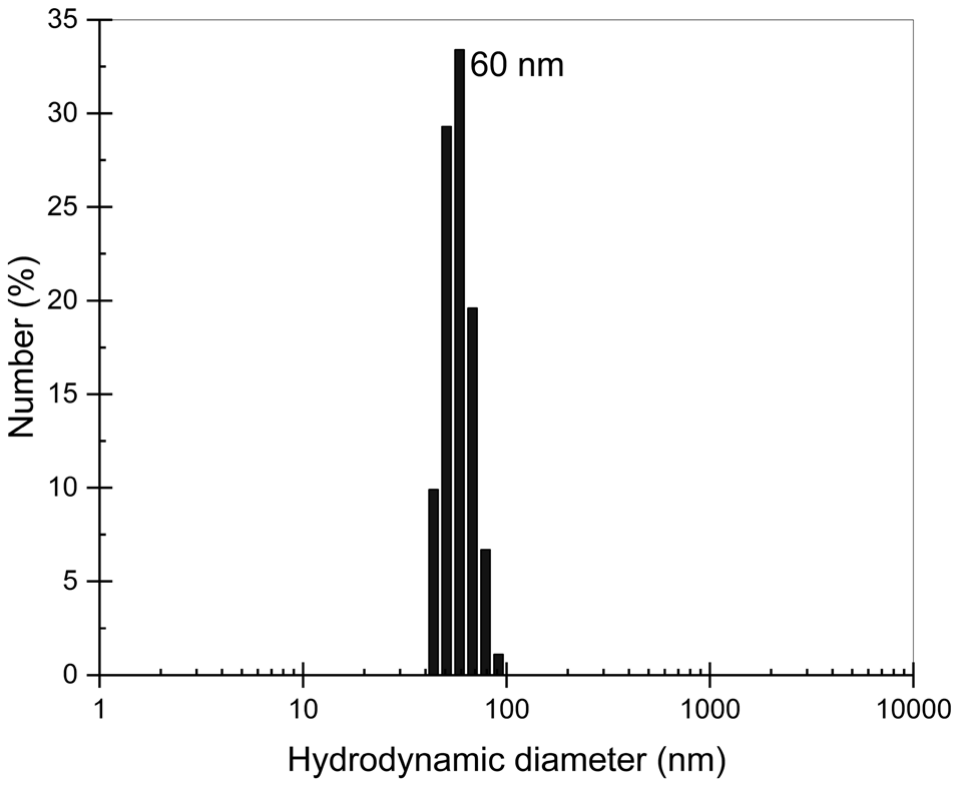
Dynamic light scattering histograms illustrating the number versus hydrodynamic diameter distribution of polymer molecules, which is in the range of 40–100 nm (average ~ 60 nm).

We used a fluorescently-labeled polymer, Poly (fluorescein isothiocyanate allylamine hydrochloride), from Sigma Aldrich in our experiments. The polymer is cationic with 56,000 Dalton molecular weight. The labeled polymer was excited at 488 nm, and emission was detected through a 540 ± 40 nm bandpass filter. The polymer powder was dissolved in distilled water to obtain a polymer solution with a concentration of 100 μg/ml. The size characterization of polymer molecules has been conducted in solution conditions. The experimental hydrodynamic size of polymer molecules in solution was determined using the dynamic light scattering method. The measurements were done by using Zetasizer Nano-ZS instrument by Malvern Panalytical with 5 s correlation time to minimize photo-induced agglomeration. The results showed a bimodal distribution of the hydrodynamic diameter, centered at about 60 nm (individual polymer molecules with almost 100% relative abundance), as shown in Fig. 3.

We performed single-phase polymer solution experiments on micromodels with the aim of capturing direct dynamic visualization of polymer entrapment. For this purpose, we used a custom-built epifluorescence microscopy setup and the fluorescently labeled polymer, which granted molecule-scale visualization. This advanced single-molecule tracking technique enabled us to observe the flow of polymer molecules within the aqueous hosting phase. The acquired grey-scale captions were post-processed in Fiji-ImageJ, and transformed into colored scale images to enhance visualization. The colored scale empathizes the different fluorescence intensity levels.

**Figure 4:**
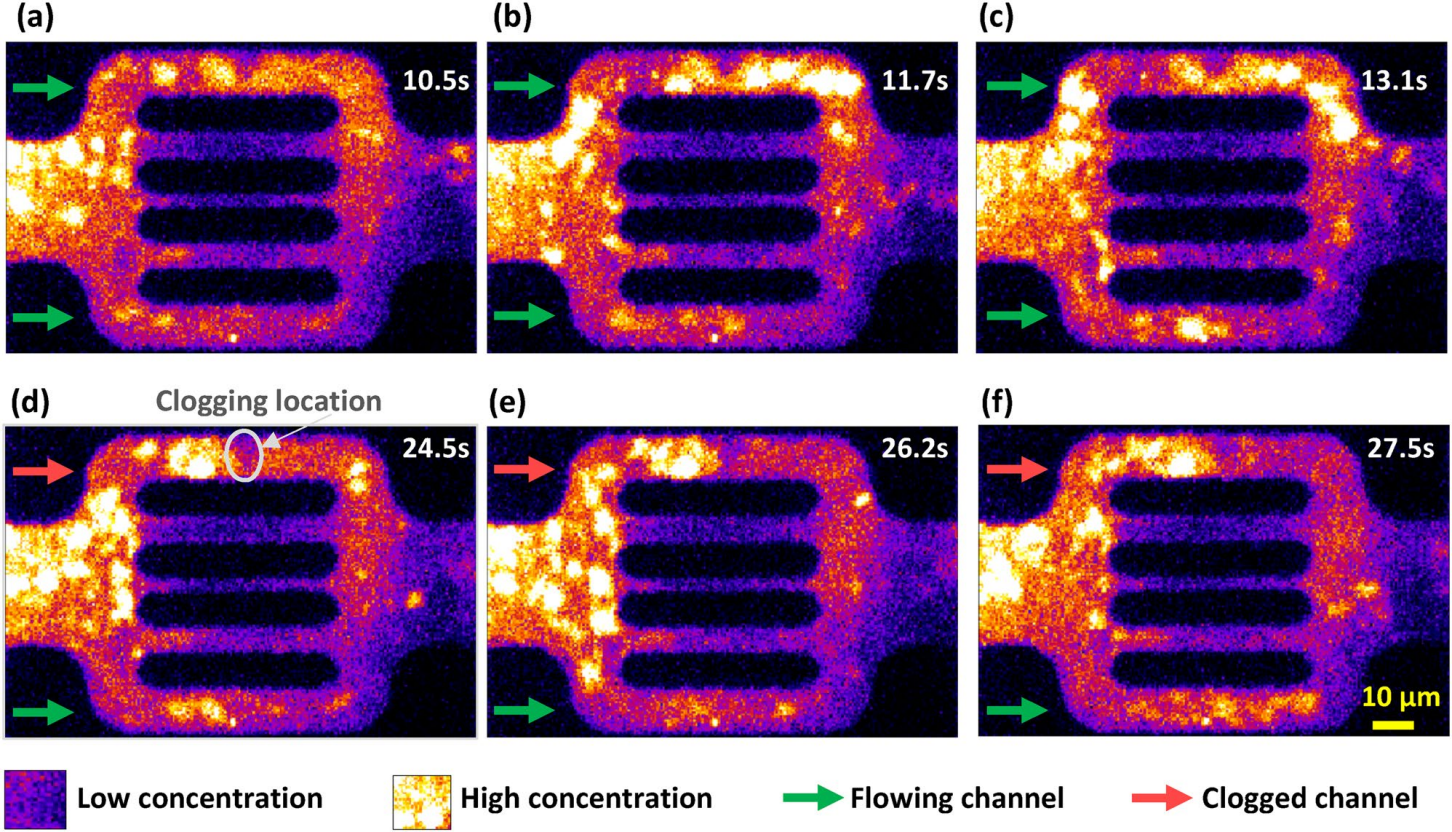
Time-lapse captions showing the flow of polymeric material reflected in bright yellow color within a flow unit, where the top row of images are taken at times 10.5, 11.7, and 13.1 s (**a**, **b**, and **c**) and exhibit open 10 µm-channels for polymer flow; the bottom row of images are taken at times 24.5, 26.2, and 27.5 s (**d**, **e**, and **f**) and exhibit mechanical entrapment of a polymer agglomerate in the top channel leading to polymer pore-clogging.

Figure 4 illustrates direct observations of polymer flooding within a flow unit captured at different times, where the bright yellow color scale reflects the presence of high fluorescent polymeric material. In contrast, the dark purple/blue color scale reflects low concentration or complete absence of polymeric material. The observed individual polymer molecules are within the 100 nm scale, while the observed agglomerations are up to 10 µm. After one hour of polymer flow through the micromodel, the middle three channels were restricted to polymeric flow. The sequence of images in the top row of Fig. 4a, b, and c show the flow behavior of the polymer within the 10 µm channels (i.e., top and bottom channels) at times 10.5, 11.7, and 13.1 s, where the reference time is 1 h after the start of polymer injection. We focus on the top and bottom 10 µm channels, where the green arrows are used to indicate open channels for fluid flow, while the red arrows indicate channel clogging. The sequence of images in the bottom row of Fig. 4d, e, and f shows when the top channel has been clogged at time = 24.5 s (d). The following images (e, f) confirm that the top channel is completely clogged, resulting in flow divergence to the bottom channels, as observed at times 26.2 and 27.5 s.

It should be noted that similar polymer flow and clogging behaviors were also observed in other flow units. The flow unit presented in this study was selected towards the middle of the microfluidic device. While some variability in flow behavior was observed across the various flow units based on their proximity to the inlet or outlet, we noted a higher degree of clogging near the inlet. This finding warrants further investigation. Nevertheless, our analysis revealed that the overall flow behavior and polymer transport were comparable across all flow units.

The observed clogging behavior is a result of the high retention of polymer. Polymer adsorption and retention at the model walls were observed at a variable thickness (~ 1-3 µm). This non-uniformity was more pronounced in the upper 10 µm channel and created a flow obstruction, reducing the area available for flow. The strangulation indicated by the circled zone in Fig. 4d favored the conditions for a bigger polymer agglomerate to get entrapment mechanically. Figure 4e–f confirm the polymer entrapment in the top channel and that the polymer/pore-size incompatibility leads to the clogging of the channel.

## Numerical method

In this study, we performed numerical simulations to predict the flow characteristics of polymer solution through a microfluidic device. The simulation work aims to replicate the flow of the aqueous phase and to compute the transport of polymer molecules prior and during pore-clogging. We used COMSOL Multiphysics® to compute the aqueous phase flow and the transport of polymer molecules. Polymer flow strongly depends on the particle size and concentration. Typical polymer concentrations for EOR applications are in the range of 500 to 3000 parts per million (ppm), at which they are regarded as dilute solutions. In some cases, lower concentrations of particles have no or insignificant effects on fluid flow, and therefore, a one-way coupling between the flow and transport, in which only the continuous host phase affects the motion of the particles and not the other way around, can be assumed. However, to account for the interactions of the polymer molecules with the medium, these assumptions do not hold. In the context of studying pore-clogging caused by polymer entrapment and its effects on the fluid flow, a full coupling is therefore needed.

We applied a two-way coupling where the interactions are bidirected so that both the fluid and the particle can affect each other^68^. The fully coupling was set between the Creeping Flow module, computing the flow of the aqueous phase, the Transport of Dilute Species module computing the flow of the polymer molecules, and the Particle Tracing module, computing the polymer agglomerates transport^68^. The coupling consists of an iterative process between the time-dependent solver for the particle trajectories, the particulate phase, and the fluid flow^68^.

### Flow governing equation

The general Navier–Stokes equation coupled with the mass conservation equation are used to model fluid flow in the micromodel, which are given by:

1 $$\rho \frac{\partial {\varvec{u}}}{\partial t}+\rho \left({\varvec{u}}\cdot \nabla \right){\varvec{u}}=\nabla \cdot [-p{\varvec{I}}+{\varvec{K}}]+{\varvec{F}}$$

2 $$\frac{\partial \rho }{\partial t}+\nabla \cdot \left(\rho {\varvec{u}}\right)=0$$

3 $$K=\mu \left(\nabla {\varvec{u}}+{\left(\nabla {\varvec{u}}\right)}^{T}\right)-\frac{2}{3}\mu \left(\nabla \cdot {\varvec{u}}\right){\varvec{I}}$$

where $\rho$ is the density of the fluid, ${\varvec{u}}$ the phase velocity field, *p* is the pressure, $\mu$ is the dynamic viscosity of the solution, and ***F*** is the sum of external body forces such as the gravity effect, which is irrelevant in our simulation because of the 2D-nature of the problem. The left-hand side of the first equation represents the inertial forces, while the right-hand side term is the sum of viscous forces, pressure forces, and external forces. The second equation represents the mass conservation equation, while in the third equation, *K* accounts for the stress tensor.

In the conducted experiments, the injection rate is very low, resulting in a Reynolds number far less than 1. Therefore, the Stokes flow governing equations are used to solve the flow of polymer solution, where inertial forces are negligible compared to viscous and pressure forces^69,70^. Moreover, the low injection rate resulted in low shear and therefore the solution did not exhibit a significant non-Newtonian effect (shear thinning). As the simulation time steps are small and the two-way coupling solves the flow equations for the updated conditions each time step, steady-state conditions for fluid flow modeling can be used within a time-step interval. Therefore, the corresponding governing equations used to compute the polymer aqueous phase flow are given as follows:

4 $$0=\nabla \cdot \left[-p{\varvec{I}}+{\varvec{K}}\right]+{\varvec{F}}$$

5 $$\nabla \cdot \left(\rho {\varvec{u}}\right)=0$$

6 $$K=\mu \left(\nabla {\varvec{u}}+{\left(\nabla {\varvec{u}}\right)}^{T}\right)-\frac{2}{3}\mu \left(\nabla \cdot {\varvec{u}}\right){\varvec{I}}$$

### Polymer molecule modeling using the Lagrangian approach

We combined two methods to simulate the behavior of particles in suspension: the Lagrangian and the Eulerian approaches. The Lagrangian approach deals with the particles individually and calculates the trajectories of each particle separately. The method is suitable for relatively large particles. In contrast, the Eulerian approach deals with the concentration of particles in the solution in the bulk phase and calculates an average representative behavior for all particles, which is suitable for relatively small particles^71^. Therefore, an explicit particle-tracking method based on the Lagrangian approach is used to model the transport of large agglomerates of polymer (> 60 nm). The simulation of particle trajectories using the Lagrangian approach is based on Newton’s equation of motion:

7 $$\frac{d\left({m}_{p}v\right)}{dt}={F}_{t}$$

where, ${m}_{p}$ is the mass of the particle, $v$ is the velocity of the particle, and ${F}_{t}$ is the total sum of forces acting on the particle. Various forces can be considered in discrete particle simulations. The characteristics of the polymer solution flowing in the microfluidics are captured by including the drag force on particle molecules^72^. The drag force on polymer molecules is caused by the difference between the velocity of the aqueous phase and the velocity of the molecules moving in the aqueous phase. Stokes drag force, including wall corrections, was used, as it is applicable for particles characterized by a relative Reynolds number much less than one. The flow mechanisms of the polymer particles are modeled by:

8 $${F}_{D}=\frac{1}{{\tau }_{p}}{m}_{p}M\left({\varvec{u}}-v\right)$$

9 $${\tau }_{p}=\frac{{\rho }_{p}{d}_{p}^{2}}{18\mu }$$

10 $$M=\frac{1}{1-\frac{9}{16}\alpha +\frac{1}{8}{\alpha }^{3}-\frac{45}{256}{\alpha }^{4}-\frac{1}{16}{\alpha }^{5}}\left(I-P\left({\varvec{n}}\right)\right)+\frac{1}{1-\frac{9}{8}\alpha +\frac{1}{2}{\alpha }^{3}}P\left({\varvec{n}}\right)$$

11 $$\alpha =\frac{{r}_{p}}{L}$$

where ${F}_{D}$ is the Stokes drag force, caused by the viscous shear stress that occurs between the fluid and the particles as the fluid flows around them, $u$ is the velocity of the carrying fluid,$v$ the velocity of the particle, ${\rho }_{p}$ is the density of the particles, ${d}_{p}$ the diameter of the particle, $\mu$ the viscosity of the carrying fluid, ${r}_{p}$ is the radius of the particle. The term *M* includes the wall correction with $L$ being the distance to the nearest wall, and $P\left({\varvec{n}}\right)$ is the projection onto the vector normal to the wall.

### Polymer molecule modeling using the Eulerian approach

The polymer molecules exhibit hydrodynamic diameter distribution center around 60 nm and the lowest and the highest values around 40 nm and 100 nm, respectively. Therefore, the assumption that the polymer molecules follow the flow of the aqueous phase holds. In consequence, for polymer molecules modeling, we adopt the Eulerian approach. This method tracks the individual particles by characterizing the particulate phase by a concentration with spatial–temporal dependency. The polymeric phase transport is modeled through diffusion and convection by computing the mass conservation equation for the phase as follows:

12 $$\frac{\partial c}{{\partial t}} + \nabla \cdot J + {\text{u}} \cdot \nabla c = 0,$$

where $c$ is the concentration of the polymeric material, ${\varvec{u}}$ the mass averaged velocity vector. The ${\text{u}} \cdot \nabla c$ term describes the convective transport due to the velocity field, and it is obtained by coupling with the Stokes flow of the aqueous phase. The mass transport due to molecular diffusion is defined by the diffusive flux vector $J$ as:

13 $$J = - D\nabla c,$$

The diffusion coefficient *D* for the polymer molecules is calculated by the Stokes–Einstein equation:

14 $$D = \frac{RT}{{N_{A} }}\frac{1}{6\pi \eta r},$$

where *R* is the gas constant, *N*_*A*_ the Avogadro’s number, T the temperature, the kinematic viscosity $\eta$ of the fluid, and $r$ the radius of the particle. The Stokes–Einstein equation is based on three assumptions that 1) the size of the diffusing particle is much larger than the solvent molecules, (2) the interactions between diffusing particles are ignorable, implying a dilute particulate phase, and (3) the shape of the diffusing particle is spherical^73^. Polymer molecules are known to have non-spherical shapes. Therefore, a deviation of the true value from the analytical value obtained by the equation is expected^74^.

### Geometry and mesh generation

**Figure 5:**
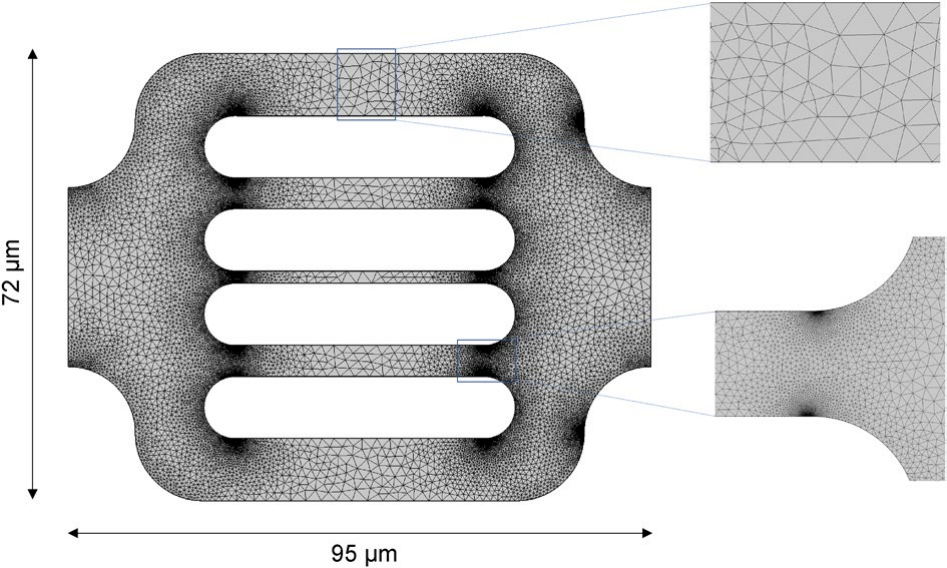
The simulation domain, reproducing the exact geometry and dimensions of a flow unit from the microdevice, and the correspondent mesh. The two magnified mesh zones correspond to the maximum element size, for the middle of the channels, and the minimum element size, for the marginal sections of the channels.

The geometry of the simulation domain was created in COMSOL by importing the E-CAD file used to fabricate the microfluidic device. Figure 5 shows the simulation domain representing a pattern of flow unit in the microdevice. The overall dimensions are 95 µm in width and 72 µm in height. The geometry consists of 5 flowing channels with apertures of 10, 5, 2, 5 and 10 µm, respectively. A finite-element unstructured triangular mesh with 60,000 elements was used, as shown in Fig. 5. The element size varies between 0.07 and 2.5 µm. This mesh resolution was found to be sufficient to minimize the gridding effect.

### Initial and boundary conditions

**Figure 6:**
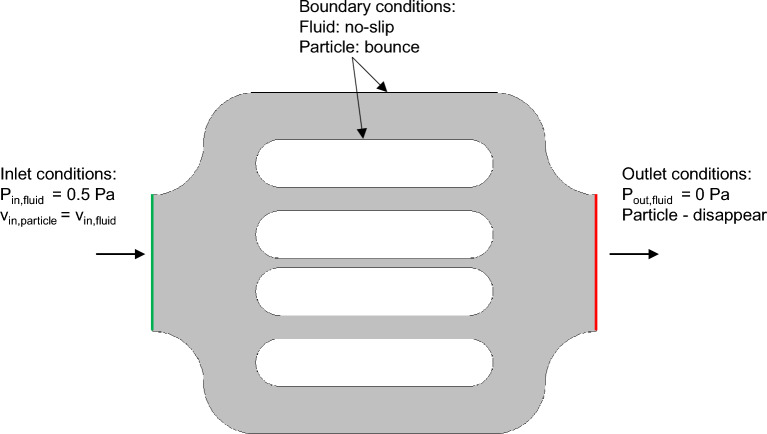
Schematic illustration of the initial and boundary conditions of the simulation domain, both for the aqueous phase and polymer molecules.

One pattern of the flow units was used in the simulation. The corresponding initial and boundary conditions, as illustrated in Fig. 6. We imposed a pressure-pressure, ${P}_{in}=0.5$ Pa and ${P}_{out}=0$ Pa respectively, at the inlet and outlet of the flow unit, representing a pressure drop of 0.5 Pa. This pressure drop was inferred from the experimental particle’s velocities. No-slip boundary conditions are set at the walls of the microdevice. The polymer concentration of 2.47 × 10^4^ mol/m^3^ was set at the inlet, based on the experimental data. The polymer particles have an inlet velocity equal to the fluid velocity at the inlet. A bounce condition was used for all walls, in which particles specularly reflect from the wall such that the kinetic energy is conserved. The aqueous phase dynamic viscosity is 2cp, corresponding to the polymer solution.

It should be noted our objective is to simulate the flow divergence and polymer clogging within one flow unit and not within the entire domain, which is not practical due to several challenges, including the computational time and the availability of observed data for the whole domain.

## Results and discussion

To replicate the flow characteristics of the polymer solution through the microdevice, a quantitative analysis of the flow and transport of polymer agglomerates through the microdevice was performed. Particle tracking velocimetry was conducted using the TrackMate functionality in ImageJ, based on the images captured during the polymer-flood experiment. The software identifies the fluorescent polymer agglomerates within the polymer solution, segments them into multiple frames, and reconstructs their corresponding trajectories. This technique can construct the flow streamlines of polymer agglomerates and calculate their transport velocities based on the particle locations, the frame number, and the frequency of captions^75^. The tested the TrackMate functionality that allows track segments to split or merge of fluorescent particles in time-lapse microscopy. The software provides the ability to split and merge tracks based on certain criteria, such as proximity or displacement, allowing for more accurate tracking of particle movements over time^76^. We found that the “*Advanced Kalman Tracker*” option is superior to the other available tracking options. Few cases were observed in our experiment which exhibited splitting and merging of the tracked particles. These cases, however, represented less than 5% of the traced streamlines and, consequently, the results were not significantly different whether the split/merge option was activated or not. The velocities vary over time depending on flow conditions and polymer-microdevice interactions. We first computed the creeping flow of the fluid phase to initialize the system. The particulate phase was then added and connected to the aqueous phase flow using full coupling.

### Stokes flow

**Figure 7:**
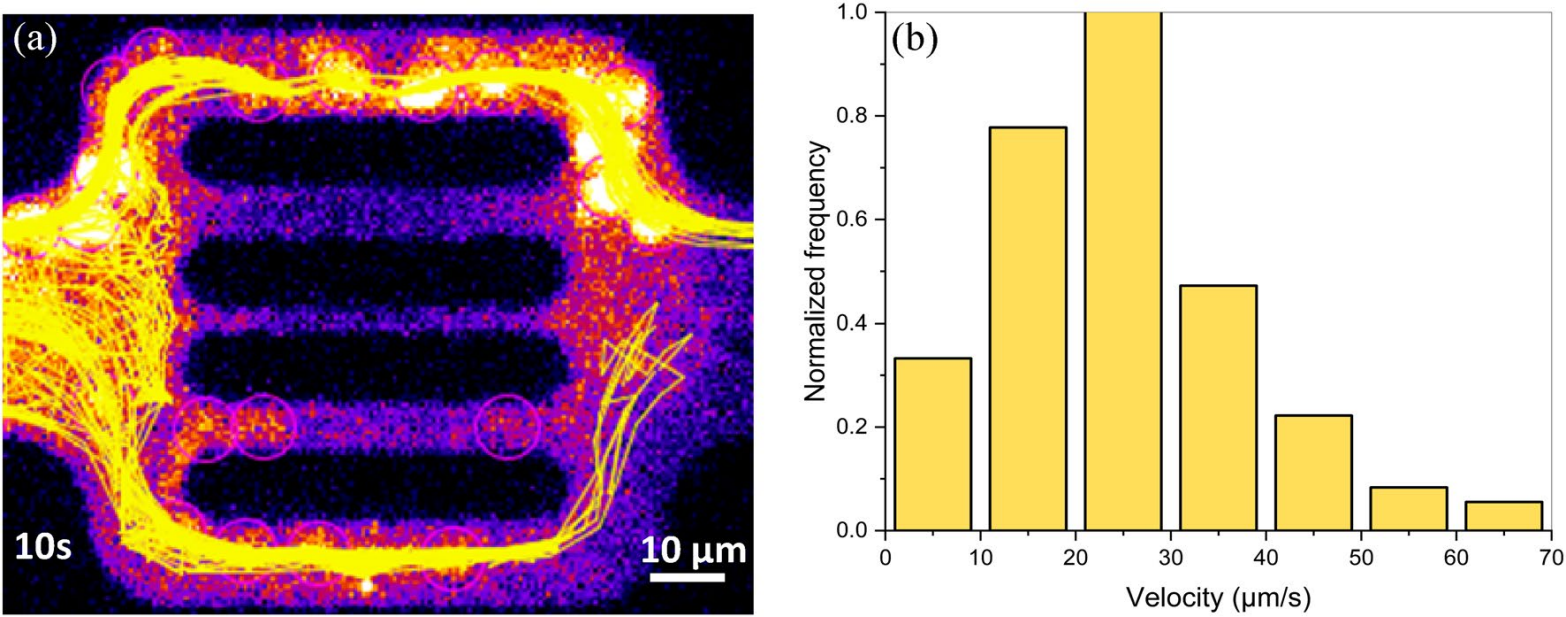
Observed polymer molecules streamlines shown in yellow during a 10 s time-interval (**a**), and the corresponding velocity histogram from experimental data (**b**).

The initialization of the Stokes fluid flow through the domain was determined based on polymer agglomerates velocity data. We measured the average velocity of the flowing polymer molecules visualized at the flow unit inlet. Figure 7a exhibits the cumulative streamlines of polymer agglomerates during 10 s of the experiment. The histogram in Fig. 7b shows the distribution of the recorded polymer molecule velocities along the streamlines. The statistical evaluation resulted in a mean velocity of around 24.6 µm/s and a rather squeezed to the left normal distribution of data with a maximum velocity less than 70 µm/s. Solving the Stokes flow using inlet velocity and outlet pressure conditions resulted in the pressure field over the entire domain.

**Figure 8:**
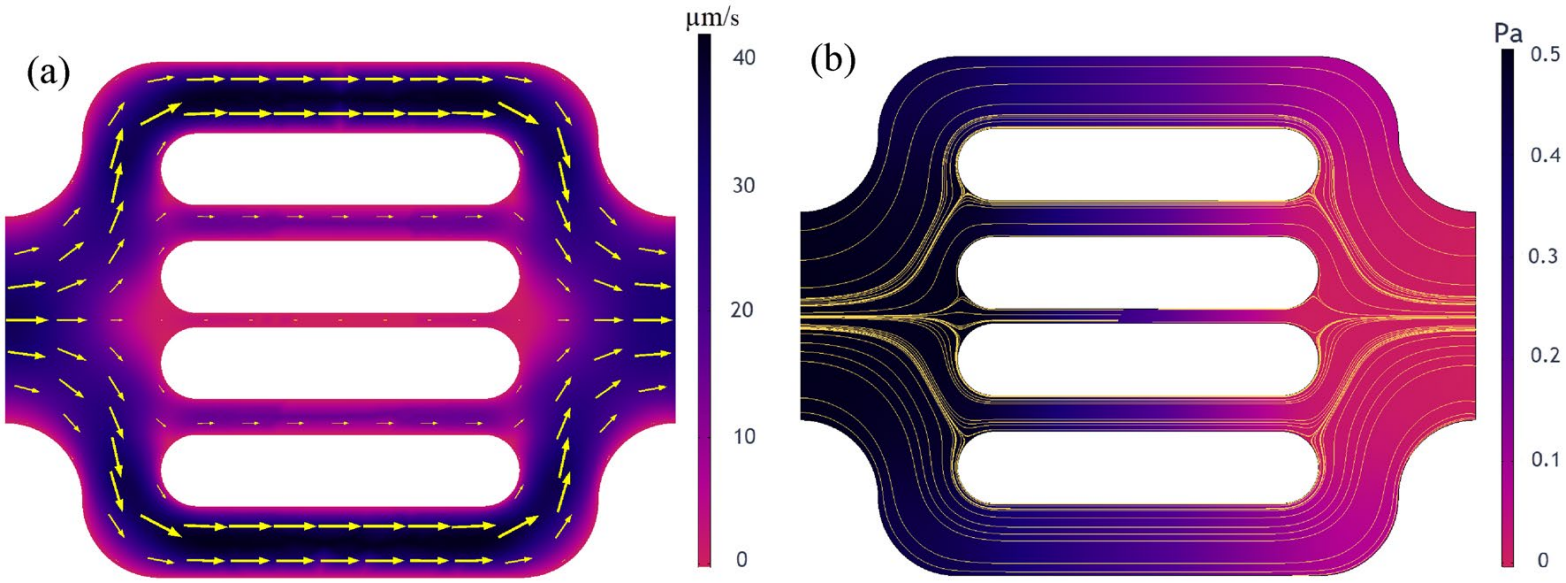
Simulation results showing the fluid flow characteristics within the simulation domain, namely the velocity field (**a**) and the pressure field with streamlines (**b**).

Besides reproducing the experimental observations, the numerical simulations offer additional insights into the flow behavior that could not be experimentally captured by the used visualization techniques. The single-molecule tracking technique attains super-resolution of hundreds of nanometers on fluorescent particles. However, it holds some several limitations^77^. Particularly on our experimental data, the high polymer retention rates on the middle three channels caused a significant reduction of flowing area and pronounced tortuosity. Due to polymer shape alteration, increased fluorescence background and photobleaching, the tortuous flow of the few polymer molecules from these channels were not clearly captured. The reduced clarity impacted polymer tracking and trajectory reconstitution during postprocessing. Figure 7a shows that the software was able to identify the polymers flowing in the middle channels (polymers encircled in the fourth channel) yet was unable to reproduce their streamlines. The parallel between experimental and simulated flow behavior from Fig. 8 highlights that numerical modeling has the capability to generate missing data and reveal velocity characteristics on the middle three channels. The yellow arrows indicate the computed flow pathways and have the size proportional to the velocity field. The numerical results exhibit velocity in the range of tens of µm/s in the 5 µm channels and of several µm/s in the 2 µm channel. The computed velocities in the whole domain range within 2 µm/s to 50 µm/s with a mean velocity of 23.6 µm/s, which is consistent with the experimental mean velocity, measured at 24.6 µm/s.

Figure 8 shows the fluid flow characteristics computed using Stokes flow*.* They correspond to the flow of the aqueous phase without including particle-medium interactions. The variable field distributions are illustrated by a color scale ranging from black, for high values, to blue, purple and red, for the low values, respectively. Figure 8a shows the velocity field within the simulation domain. As expected, the 10 µm channels exhibit less resistance to flow, resulting in the highest velocities with values within 30 µm/s. On the other hand, the 2 µm channel experiences the lowest velocities, with values within 8 µm/s. In parallel, Fig. 8b shows the pressure field and the streamlines, in yellow, within the simulation domain.

### Polymer agglomerates—Lagrangian model

**Figure 9:**
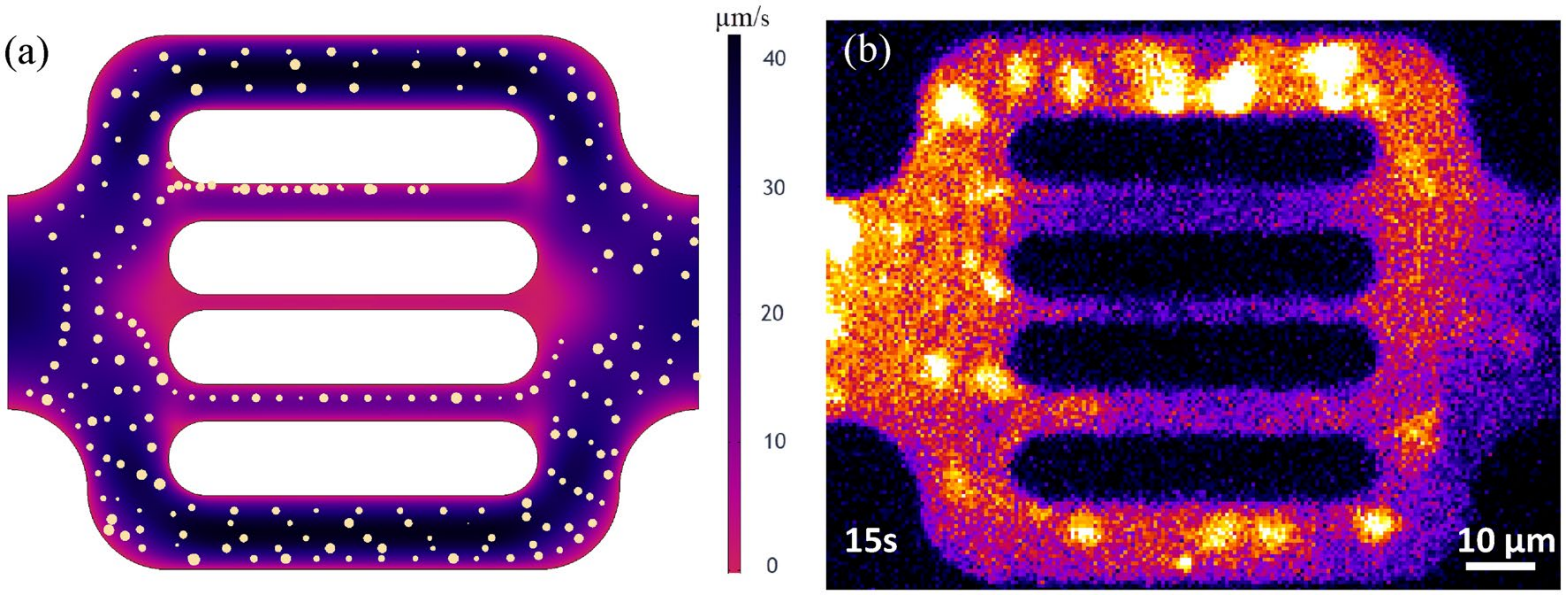
Numerical modeling of polymer molecules flow, shown as yellow particles, and the corresponding fluid velocity field, colored background (**a**), and an actual caption of polymer molecules, shown in bright yellow, flow through the microdevice during experiments (**b**).

The polymer’s agglomerates flow paths are computed using the Particle Tracing module, where the agglomerates are simulated as solid particles initiated as a normal particle size distribution ranging between 1.2 and 6.5 µm with 4 µm on average. This range replicates the experimentally measured size distribution of the polymer agglomerates in the solution. Particle initialization required four main parameters, including initial position, initial velocity, release time, and the number of particles. The initial position of the particles is set at the inlet of the flow unit, distributed along the boundary proportionally to the magnitude of the fluid velocity field. The initial velocity of the particles corresponds to the inlet flow velocity. The particles were released in slugs of 10 particles at a time at 0.3 s intervals. Figure 9 shows both the computed and the experimentally captured polymer transport within the hosting aqueous solution for a flow unit that did not exhibit polymer clogging. Figure 9a depicts the results of the numerical modeling of polymer flowing agglomerates within the domain, while Fig. 9b shows the experimental observations. The model could qualitatively mimic the flow of large polymer agglomerates but does not capture the overall concentration of polymer; thus, an Eleurian approach is needed.

### Combined Eulerian–Lagrangian model

**Figure 10:**
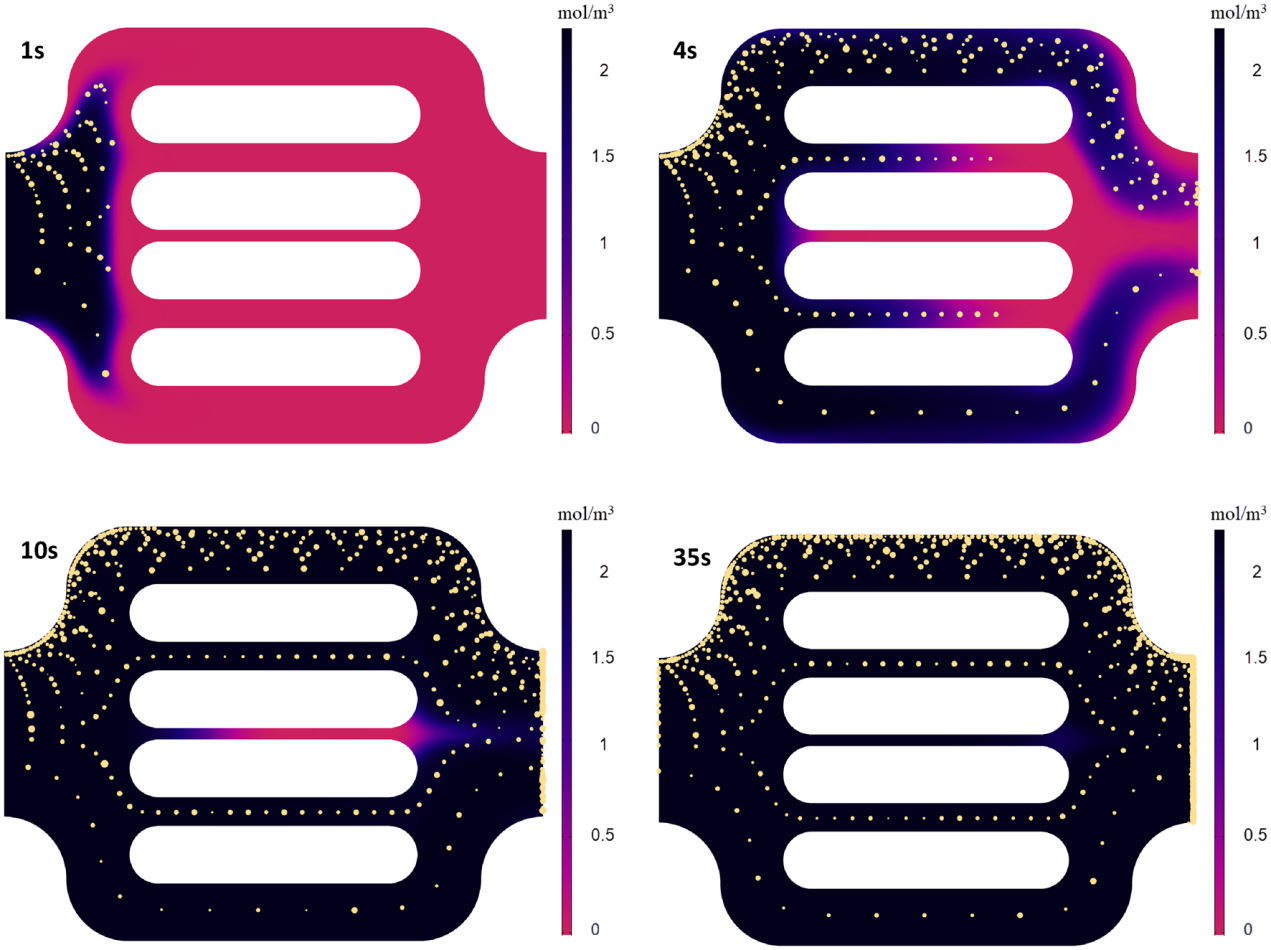
Simulation results showing polymer concentration (mol/m^3^) at different times, and the transport of polymer agglomerates, characterized by discrete particles.

We introduced the polymer molecules and computed their flow using the Transport of Dilute Species module in COMSOL, where the polymer is tracked with an advection–diffusion model. The experimentally measured size distribution of the hydrodynamic diameter of the single polymer molecules was centered around 60 nm, with 40 nm and 100 nm being the lowest and the highest value, respectively. The mean value, 60 nm, was used to analytically calculate the diffusion coefficient using the general Stokes–Einstein equation, which provided a value of 1.6 × 10^−12^ m^2^/s. Figure 10 shows the flow behavior of both polymer molecules and polymer agglomerates within the flow unit without accounting for any particle-medium interactions. The discrepancy between the front of the polymer molecules and polymer agglomerates is caused by the underlying physics that governs the flow. The drag force on the individual polymer agglomerates acts in the opposite direction of the flow, inducing retardation of the polymer agglomerates compared to the flow field. On top, diffusive forces included in modeling the polymer molecules' phase act in the same direction as the flow field. However, due to the significant difference in magnitudes between the viscous and the diffusive forces, the effect of the diffusion on the streamlines is negligible. The contribution of diffusive is observed in the near-wall region with null velocity due to the non-slip condition. The initial breakthrough of the polymer molecules is after 4 s, and the steady-state is achieved at 28 s, when the concentration within the unit is constant and equal to the inlet value of 2.47 × 10^5^ mol/m^3.^

### Polymer-pore clogging

We modeled the flow alteration induced by polymer entrapment that was observed during the experimental study by considering polymer-medium interactions. However, polymer mechanical entrapment in porous media lacks a predictive model. Alternatively, we developed a clogging model that behaves as a porous screen through which only a specified size of polymer molecules can go through while the rest are entrapped and accumulated at the screen, which results in conductivity reduction. To replicate the polymer’s mechanical entrapment observed in the experiment, screen models were introduced in the channels. The fluid flow resistance through the screen is captured by a resistance factor which is a function of the number of entrapped polymer agglomerates. With this approach, when a particle hits the screen and meets the conditions of size exclusion, the resistance coefficient is incremented, and therefore it captures the cumulative particle-screen interactions. This clogging model, however, does not completely clog water flow by convection and diffusion, which is consistent with the inaccessible pore-volume (IPV) concept, where large polymer molecules cannot reach tight pore bodies while water can^78^.

**Figure 11:**
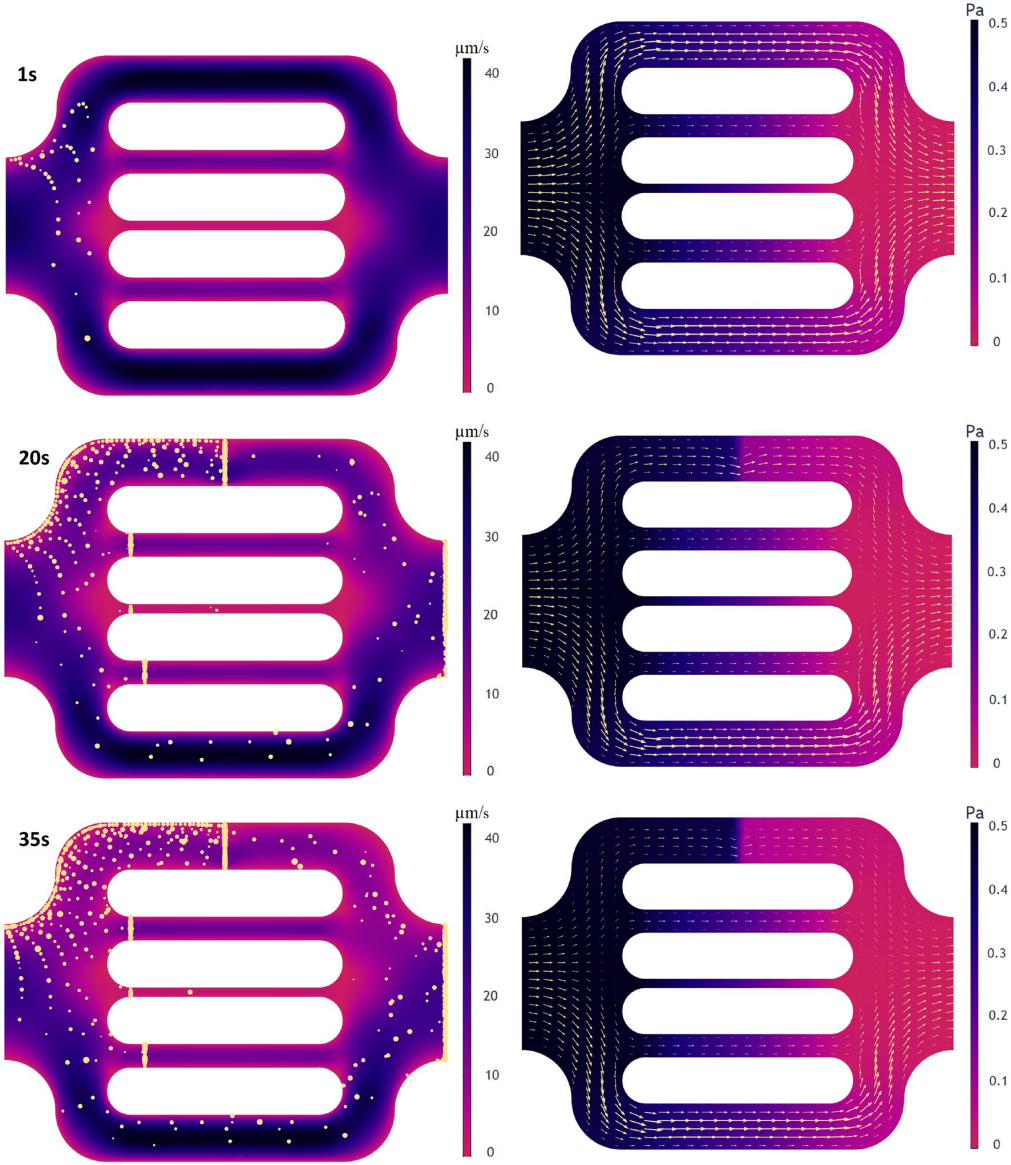
Simulation results for flow characteristics showing polymer clogging at different times; the map of velocity magnitude and particle transport and entrapment are shown on the left, while the pressure field with velocity arrows scaled by velocity magnitude are shown on the right.

Figure 11 shows the computed flow characteristics during numerical simulating clogging. The entire simulation time was 35 s, similar to the experimental observation time of the particular flow unit where polymer mechanical entrapment was captured.

Figure 11 illustrates the velocity field and the transport of polymer particles (left side). On the right-hand side, the pressure field is represented by the velocity field with arrows scaled by velocity magnitude at three different times, 1, 20, and 30 s. At early time, 1 s, few particles were released but have not yet reached the clogging screens. It can be observed that the flow behavior remains unchanged, and its characteristics are similar to the unaltered flow described in Fig. 8. The snapshot from the second row corresponds to the intermediate simulation time, 20 s, during which a significant number of particles flew through the unit. The flow characteristics exhibit a noticeable reduction of flow within the top channel. The velocity magnitude reduced from higher than 40 µm/s to approximately 25 µm/s. On the other hand, the pressure, with initial uniform variation, is characterized by a sharp change across the screen (0.5 Pa to 0.1 Pa), induced by decreased connectivity due to clogging. The flow velocity suggests that the model was able to create the flow obstruction on the upper part of the bottom channel, and closely simulate the experimental observations with the flow mainly happening on the bottom of the channel. The last row exposes the flow characteristics in the last time step. The velocity on the top channel exhibits a more significant reduction caused by increased clogging effects. There is a further reduction from 10 to 25 µm/s with a significant pressure drop across the screen were obtained.

**Figure 12:**
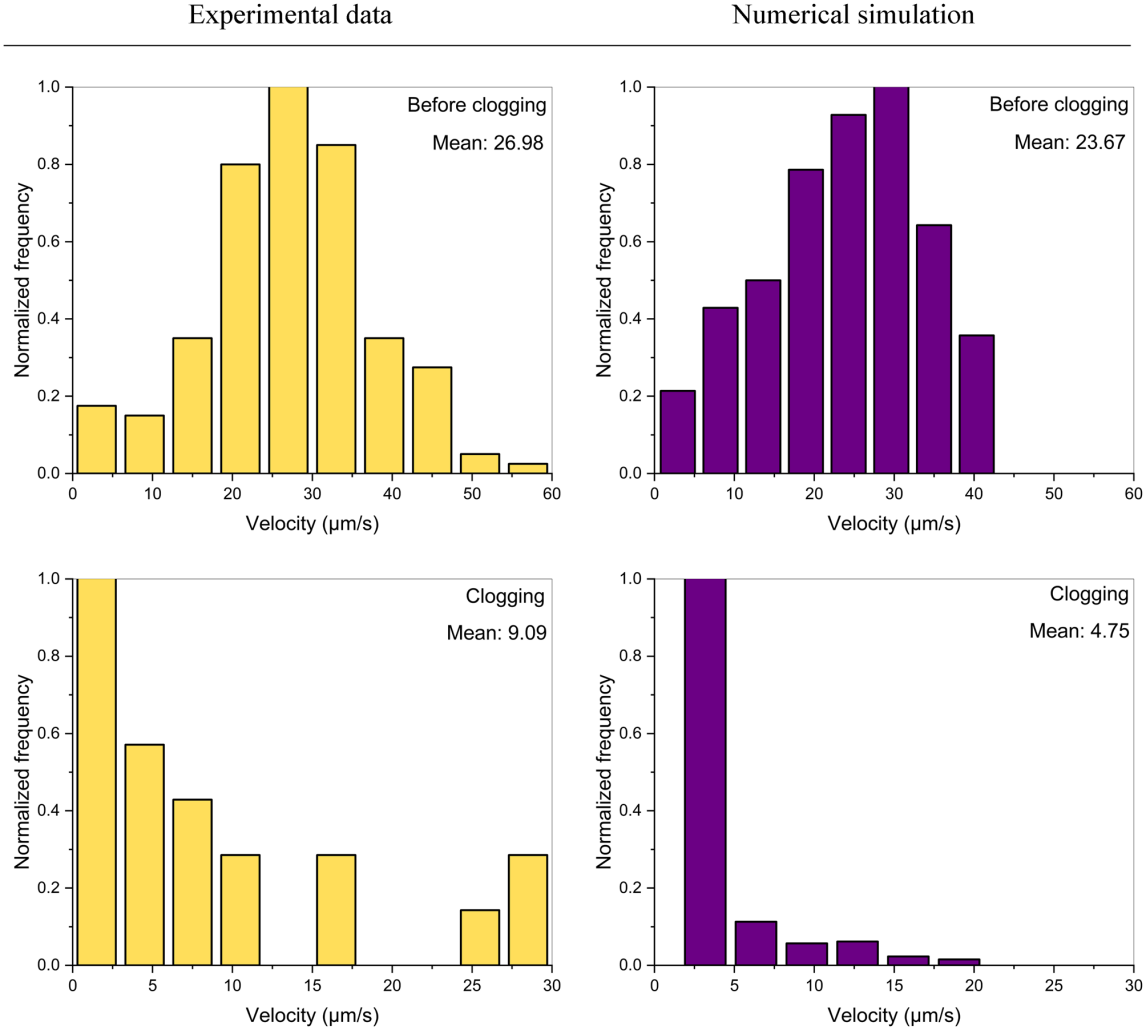
Velocity histogram of polymer molecules from experimental results (left), and the velocity histogram of the particles from simulations (right) before and during polymer-induced pore-clogging.

We performed a quantitative analysis of the fluid flux across the top channel to obtain a deeper understanding of the flow characteristic before and during pore-clogging. The analysis was performed both on experimental and simulated data, investigating the flow velocity of polymer molecules and particles, respectively. The streamlines and velocity of polymer molecules from the experimental observation were determined using the particle tracking functionality in Fiji. Figure 12 shows the velocity of polymer molecules in the first column, in yellow, and the computed velocity of the particles in the second column, in purple, for two-time intervals: before and during clogging. Figure 12-first row illustrates the velocity histograms of polymers(left) and particles(right) before clogging. The computed velocities capture the value interval of the most predominant polymer molecule velocities, up to 40 µm/s. The computed velocities distribution resembles a Gaussian distribution of discrete experimental data, with the peak velocity values between 20 and 30 µm/s. The obtained mean velocity of 23.67 µm/s closely replicates the mean polymer velocity of 26.98 µm/s. Figure 12-second row shows the velocity histograms of polymers and particles (right) during clogging. The computed particle velocities honor the main velocity interval of polymers, with values lower than 20 µm/s. The computed velocities distribution resembles a Gaussian distribution of discrete experimental data, with the peak velocity values lower than 5 µm/s. The data highlight a reduction of 66% in mean velocity during polymer entrapment. The agreement in mean velocities substantiates that the numerical model is able to mimic the flow characteristic before and during polymer-pore clogging experimental results.

## Conclusions

We presented an experimental and numerical study of polymer solution flow through a microfluidic device, which captures the effect of polymer clogging at the pore scale. The objective is to study the effect of pore-scale polymer flow behavior altered by polymer-medium interactions and the changes in flow characteristics during polymer mechanical entrapment and pore-clogging.

The key conclusions are summarized as follows:

- The proposed microfluidics technology coupled with a microscope setup allowed direct observations of polymer retention at pore- and molecule-scale under dynamic conditions.
- The numerical model accurately imitates the observations of the fluid flow behavior unaltered by polymer retention and replicates the flow paths and velocity of both the aqueous and particulate phases before clogging.
- Polymer mechanical entrapment was simulated along with the implicit flow behavior modification. The velocity within the channel reduced from values higher than 40 µm/s before clogging to values lower than 15 µm/s during clogging. The pressure with initial uniform variation is characterized by a sharp change across the screen during clogging, of 0.5 Pa on one side and less than 0.1 Pa on the other side.
- The results showed significant flow characteristic changes during polymer-pore clogging. The velocity and pressure field, as well as streamlines alteration, substantiate that the generated numerical model replicates the flow behavior experimentally observed during pore-clogging.
- The results of the quantitative analysis of polymers/particle flow showed a 66% reduction in velocity during clogging, from mean values around 27 µm/s before clogging to around 9 µm/s during clogging. Moreover, the numerical model accurately predicts the flow parameters, with emphasis on velocity, before and during polymer-induced pore-clogging.
- The proposed experimental–numerical approach offers additional insights into polymer flow behavior at the pore scale.

## Supplementary Information

Supplementary Information 1.

Supplementary Video 1.

## Data Availability

The data used in this study is available within the article that support the findings of the present research work.
