# Supplementary material for: Experimental and numerical investigation of polymer pore-clogging in micromodels
Source: Sci Rep. 2023 May 22;13:8245. doi: 10.1038/s41598-023-34952-9 (PMC10203338; doi:10.1038/s41598-023-34952-9)
Supplement: Supplementary file 1 — Supplementary Information 1. [file 41598_2023_34952_MOESM1_ESM.docx]

**Supporting Information**

Experimental and Numerical Investigation of Polymer Pore-Clogging in Micromodels

Antonia Sugar^1^, Maged Serag^2^, Ulrich Buttner^3^, Marwan Fahs^4^, Satoshi Habuchi^2^, Hussein Hoteit^1*^

^1^Physical Science and Engineering Division,

King Abdullah University of Science and Technology (KAUST), Thuwal, Saudi Arabia

^2^Biological and Environmental Science and Engineering Division,

King Abdullah University of Science and Technology, Thuwal, Saudi Arabia

^3^Nanofabrication Core Lab,

King Abdullah University of Science and Technology, Thuwal, Saudi Arabia

^4^Laboratoire d’Hydrologie de Geochimie de Strasbourg, University of Strasbourg, CNRS, France

*Corresponding author: Hussein.hoteit@kaust.edu.sa

**Brief video titles and legends for the supplementary video:**

Video_S1: Video showing the flow of polymeric material reflected in bright yellow color within a flow unit, consisting of 5 channels with dimensions 10 µm, 5 µm, 1µm, 5 µm, 10 µm, respectively, from top to bottom. The video runs for about 100 seconds, which exhibits a mechanical entrapment phenomenon of a polymer agglomerate in the top channel after about 60 seconds, leading to total polymer pore-clogging of the top channel.
